# Collaborative Optimization of Density and Surface Roughness of 316L Stainless Steel in Selective Laser Melting

**DOI:** 10.3390/ma13071601

**Published:** 2020-04-01

**Authors:** Yong Deng, Zhongfa Mao, Nan Yang, Xiaodong Niu, Xiangdong Lu

**Affiliations:** 1 Intelligent Manufacturing Key Laboratory of Ministry of Education, Shantou University, Shantou 515063, China; dengyong@stu.edu.cn (Y.D.); nyang@stu.edu.cn (N.Y.); 15xdlu@stu.edu.cn (X.L.); 2 Digital Technology Research and Application Center, Shantou Polytechnic, Shantou 515078, China; 3 Shantou Ray-Bonus Additive Manufacture Research Institute, Shantou 515063, China

**Keywords:** selective laser melting, 316L stainless steel, multi-objective optimization, relative density, surface roughness

## Abstract

Although the concept of additive manufacturing has been proposed for several decades, momentum in the area of selective laser melting (SLM) is finally starting to build. In SLM, density and surface roughness, as the important quality indexes of SLMed parts, are dependent on the processing parameters. However, there are few studies on their collaborative optimization during SLM to obtain high relative density and low surface roughness simultaneously in the literature. In this work, the response surface method was adopted to study the influences of different processing parameters (laser power, scanning speed and hatch space) on density and surface roughness of 316L stainless steel parts fabricated by SLM. A statistical relationship model between processing parameters and manufacturing quality is established. A multi-objective collaborative optimization strategy considering both density and surface roughness is proposed. The experimental results show that the main effects of processing parameters on the density and surface roughness are similar. We observed that the laser power and scanning speed significantly affected the above objective quality, but the influence of the hatch spacing was comparatively low. Based on the above optimization, 316L stainless steel parts with excellent surface roughness and relative density can be obtained by SLM with optimized processing parameters.

## 1. Introduction

Selective Laser Melting (SLM) is an additive manufacturing (AM) technology based on use of a high-power laser beam, and is the most widely used metal 3D printing technology [1]. SLM manufacturing is a rapid prototyping process, in which metal powder is melted layer by layer and then solidified to form parts. Compared with conventional manufacturing processes, SLM has many outstanding advantages, such as the capacity to manufacture parts with complex structures, saving time and costs [1,2].

However, there are more than 130 parameters [3] of SLM which may have impacts on the forming properties (such as density, surface roughness (defined as SR) and thermal properties [4]) of the parts, including the diameter of the laser beam, laser power (defined as P), scanning speed (defined as V), hatch spacing (defined as S), scanning strategy, layer thickness and so on [5,6,7,8]. Mumtaz [9], Song [10] and Dadbakhsh [11] investigated the effects of processing parameters including laser power and scanning speed on surface roughness, and revealed that higher laser power tended to reduce top surface roughness. Han et al. [12] studied the factors influencing the surface roughness in SLMed AlSi10Mg parts, and found that surface roughness was affected mainly by the scanning speed and hatch spacing. Larimian et al. [13] investigated the effect of the scanning strategy on the density of 316L stainless steel SLMed parts and indicated that higher scanning speeds had better densification. Ni et al. [14] investigated the density and mechanical properties of SLMed Hastelloy X Alloy parts of processing parameters, and found that the relative density increased slightly with increasing laser power or decreasing scanning speed in the printing processes.

To obtain SLMed parts of a higher quality, research on the processing parameters optimization in SLM has been conducted. Wang et al. [15] proposed an orthogonal method to optimize the processing parameters and obtained a higher density of ASTM A131 (EH36 grade) steel parts. They found that the density showed a nearly linear relationship with the scanning speed, i.e., the lower the scanning speed, the higher the density. Song et al. [16] and Wang et al. [17] simulated the temperature distribution and optimized the laser scan speed using the finite element analysis (FEA) method, and obtained higher density Ti6Al4V alloy parts at a laser power of 110 W and scan speed of 0.2 m/s. Li et al. [3] employed response surface methodology (RSM) to optimize the SLM parameters for better surface roughness of Ti6Al4V alloy parts, and proved that the response surface method was effective in SLM parameters optimization.

The above research mainly focused on the influences of single or multiple processing parameters on the surface roughness or density of SLMed parts, respectively. Few studies address the synergistic processing optimization on both roughness and density for 316L stainless steel by using the RSM method in the literature.

In this study, the influence of laser power, scanning speed and hatch spacing on density and surface roughness are studied. A relationship model of processing parameters and forming quality is established. As a unique contribution, a multi-objective collaborative optimization strategy based on RSM is proposed for obtaining good surface roughness and density simultaneously.

## 2. Materials and Methods

### 2.1. Experimental Materials

**Figure 1:**
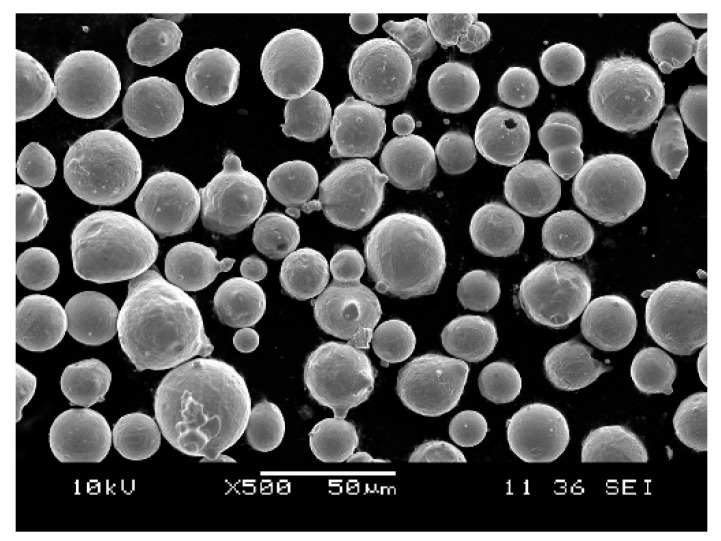
The morphology of the raw powder material.

The raw material used in this study is 316L stainless steel powder (Hunan Farsoon High-Technology Co., Ltd., Hunan, China). This material, as one of the most commonly used stainless steels, has excellent corrosion resistance and high temperature resistance, and has been widely used in the construction, petrochemical and food industries [1,18]. As shown in Figure 1, the powder morphology is almost spherical with some small satellite particles, which is favored as it improves the flowability and distribution of the powder on the powder bed [19], and thus helps to obtain a good quality of finished parts.

### 2.2. Experimental Equipment and Method

The SLM equipment employed in this study was an FS271M metal 3D printer (Hunan Farsoon High-Technology Co., Ltd., Hunan, China); its specifications and parameters are shown in Table 1. Experimental samples with the size of 8 mm × 8 mm × 8 mm were fabricated on the base plate of this equipment and removed by wire cutting. Surface roughness was measured using a TR200 roughness meter with an accuracy of 0.001 μm (Beijing Jitai Scientific Instrument & Testing Equipment Co., Ltd., Beijing, China) using a contact measurement method [20]. First, the bottoms of samples were polished to a plane. Then, five different positions on the top surface of samples were selected for surface roughness measurement, and these measuring values were averaged. The relative density (defined as RD) is expressed as the ratio between the measured density and the theoretical density. The measured density was acquired by a high-precision BSM-220.4 electronic balance (Shanghai Zhuojing Electronic Technology Co., Ltd., Shanghai, China) with an accuracy of 0.0001 g using the Archimedes method [21]. Before density measurement, all surfaces of the samples were polished with 1000-grit abrasive paper to obtain more accurate measurement results. The effective measurements were conducted at least five times and their measurement values were averaged. The density formula of the part is shown in Equation (1).

(1) $$\rho =\frac{m_{1}}{m_{1}-m_{2}}\rho_{o}$$

where ρ represents the density of the sample; $m_{1}$ represents the mass of the sample measured in air; $m_{2}$ represents the mass of the sample measured in water; and $\rho_{o}$ represents the density of distilled water.

### 2.3. Experimental Design

Response Surface Methodology (RSM) is a method of expressing the influences of multiple factors on the response by constructing a polynomial [22,23,24]. The objective is to find an optimal combination of factors that have an excellent response. RSM has been widely used and has been proved to be an effective optimization method [3,22,23,24]. One outstanding advantage is that RSM gives the mathematical expression of the process, which not only reveals the relationship between factors and responses, but also includes the interaction between factors [3]. Therefore, RSM has been used to optimize SLM process parameters to produce higher quality parts [3,22]. The second-order polynomial response surface model [23] is shown in Equation (2).

(2) $$Y=\beta_{0}+\overset{k}{\underset{i=1}{\sum }}\beta_{i}x_{i}+\overset{k}{\underset{i=1}{\sum }}\beta_{ii}x_{i}^{2}+\overset{k}{\underset{i<j}{\sum }}\beta_{ij}x_{i}x_{j}+\varepsilon$$

where, Y represents the response, $x_{i}$ represents the input factors, $\beta_{i},\;\beta_{ii},\;\beta_{ij}$ are undetermined coefficients, $\beta_{0}$ is the mean and ε is the error.

Central Composite Design (CCD), the most widely used method of RSM [24], was selected to design the experimental scheme. The input factors in this experiment are laser power, scanning speed, and hatch spacing. The three parameters of each input factors correspond to different levels. Each factor is coded at five levels, as shown in Table 2.

## 3. Results

### 3.1. Main Effects of Processing Parameters

The experimental scheme, with 20 groups of experiments, was designed in Minitab, a data analysis, predictive analysis and process improvement software package, from which we can create the experimental design matrix, analyze the results, and predict the responses. The experimental results were measured, as shown in Table 3. It can be seen that the optimal values of the density and the surface roughness in these samples can be obtained respectively when using the processing parameter sets (P, V, S) of NO.11 (225 W, 700 mm/s, 90 μm) and NO.19 (225 W, 1000 mm/s, 90 μm).

**Figure 2:**
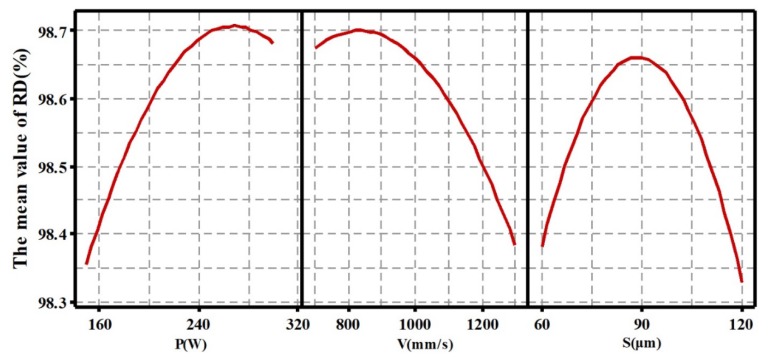
Main effects of processing parameters on relative density (RD).

**Figure 3:**
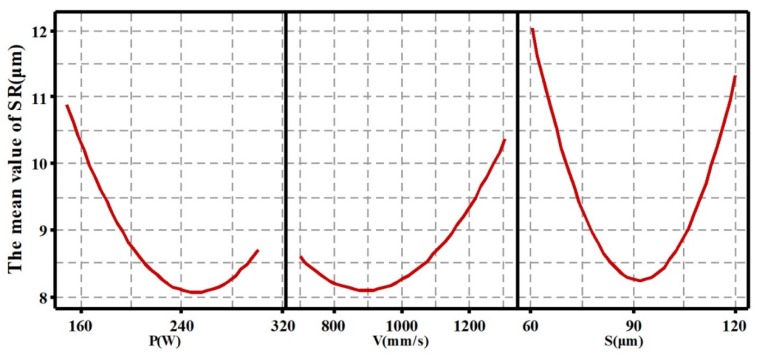
Main effects of processing parameters on surface roughness (SR).

The main effects of processing parameters on RD and roughness are shown in Figure 2 and Figure 3. From Figure 2 and Figure 3, it can be observed and concluded that: (1) With the laser power increasing in the range of 150–300 W, the RD of the SLMed parts first increases and then decreases. The maximum value occurs at a P of near 270 W. On the contrary, the SR first decreases and then increases. The minimum value appears at a P of near 250 W. (2) Similarly, the RD first increases and then decreases with the increase of scanning speed from 700 mm/s to 1300 mm/s, and reaches the maximum value at a V of near 840 mm/s; however, the SR first decreases and then increases, the minimum value appears at a V of near 900 mm/s. (3) For different S, as the S increases from 60 μm to 120 μm, the RD first increases and then decreases, and the surface roughness first decreases and then increases. Their corresponding maximum/minimum values both occur around the S of 90 μm.

**Figure 4:**
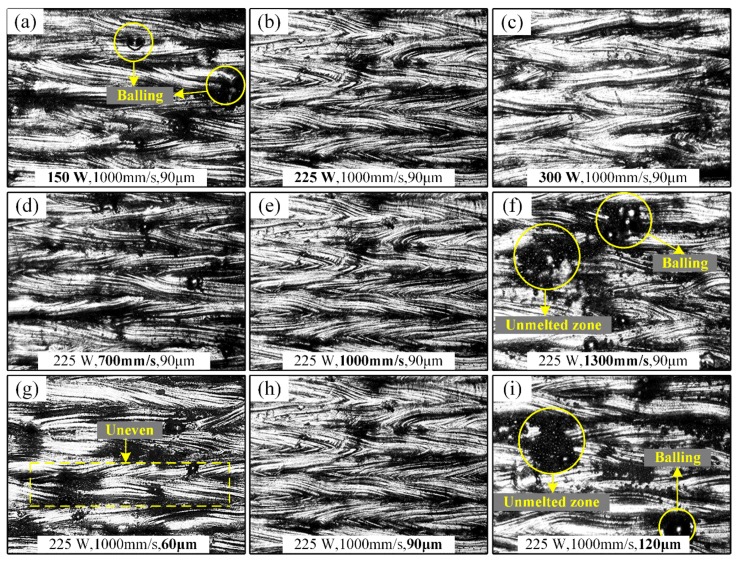
Surface morphologies of parts with different P values of (**a**) 150 W, (**b**) 225 W and (**c**) 300 W, and different V values of (**d**) 700 mm/s, (**e**) 1000 mm/s and (**f**) 1300 mm/s, and different S values of (**g**) 60 μm, (**h**) 90 μm and (**i**) 120 μm.

In order to better compare the effects of processing parameters on SR, some samples in Table 3 were chosen to observe their morphologies, as shown in Figure 4. It is noted that Figure 4b,e,h came from the same sample as No. 17 in Table 3. Under the premise of keeping the other processing parameters constant, surface morphologies of samples (Nos. 9, 17, 10) with different P values (150 W, 225 W, 300 W) are shown in Figure 4a–c. In Figure 4a, when the P is 150 W, a balling phenomenon on the surface of the sample is obvious and is unevenly distributed. When the laser power increases to 225 W in Figure 4b, a clear scaly feature can be observed, and homogeneous molten pool tracks contribute the excellent SR value of 8.06 μm. However, with the increase of P, surface quality (Figure 4c) is worsened. These features are consistent with main effect of P (Figure 3).

For different values of V (700 mm/s, 1000 mm/s, 1300 mm/s), surface morphologies of samples (Nos. 11, 17, 12) are shown in Figure 4d–f. It is interesting that there are few changes in surface morphology with the V increasing from 700 mm/s to 1000 mm/s, but morphology begins to dramatically worsen as the V further increases. When the V increases to 1300 mm/s in Figure 4f, the balling phenomenon becomes obvious and some unmelted zones on the surface of the sample are clearly visible. These features directly contribute to the deterioration of the surface quality, and simultaneously lead to the obvious reduction of the RD.

For different values of S (60 μm, 90 μm, 120 μm), surface morphologies of samples (Nos. 13, 17, 14) are shown in Figure 4g–i. When S is 60 μm, the uneven morphology (Figure 4g) on the single track can be clearly seen, confirming the unstable molten pool phenomenon. As S increases to 90 μm, the morphology becomes smoother and more uniform, as shown in Figure 4h. However, when S further increases to 120 μm, unmelted zones and balling (Figure 4i) begin to occur on the surface, proving that the laser energy supplied is not enough to melt the powder.

### 3.2. Analysis of Variance

Based on measured data (Table 3), analysis of variance (ANOVA) was carried out, and the quadratic response surface models for RD and SR were built using Minitab software. These models reflect the mathematical relationships between RD/SR and processing parameters respectively, as presented in Equations (3) and (4).

(3) $$\begin{array}{c} \mathrm{RD}=90.677+0.01794\;P\;+0.003029\;V\;+0.09969\;S\;-0.000025{\;P}^{2}\\ \;-0.000001{\;V}^{2}-0.000332{\;S\;}^{2}+0.000004\;P*V\;-0.000101\;P\\ \;*S\;-0.000018\;V*S \end{array}$$

(4) $$\begin{array}{l} \mathrm{SR}=82.82-0.1291\;P\;-0.04462\;V\;-0.8227\;S\;+0.000268{\;P}^{2}+0.000013{\;V}^{2}\\ \;+0.003730{\;S}^{2}+0.000015\;P*V\;-0.000234\;P*S\;+0.000192\;V\\ \;*S \end{array}$$

Here, P*V, P*S and V*S mean two-factor interaction effects.

The results of ANOVA for RD and SR are given in Table 4. The F test was used to determine the significance of the various input factors. For the linear effects in Table 4, it was found that the influences of processing parameters have the same significance of influences on the RD and the SR are similar. The *p*-values of P and V are both less than 0.01, which indicates that their influences both on the RD and the SR are highly significant. The *p*-value of S is less than 0.25 and greater than 0.05, revealing that it has a weak influence. As for two-factor interaction effects, there are some differences between the RD and the SR. For the RD, P*V with the *p*-value (0.043) has a significant effect. Furthermore, *p*-values of P*S and V*S are both less than 0.01, indicating that they have highly significant effects on the RD. However, for the SR, only V*S with the *p*-value (0.001) presents a significant effect. In addition, it can be seen in Table 4 that the models fit the experimental data well with R^2^ values of 96.65% and 96.54%, respectively. The corresponding prediction values R^2^ are 80.26% and 79.77%, meaning that the models can effectively predict the RD and SR of parts.

**Figure 5:**
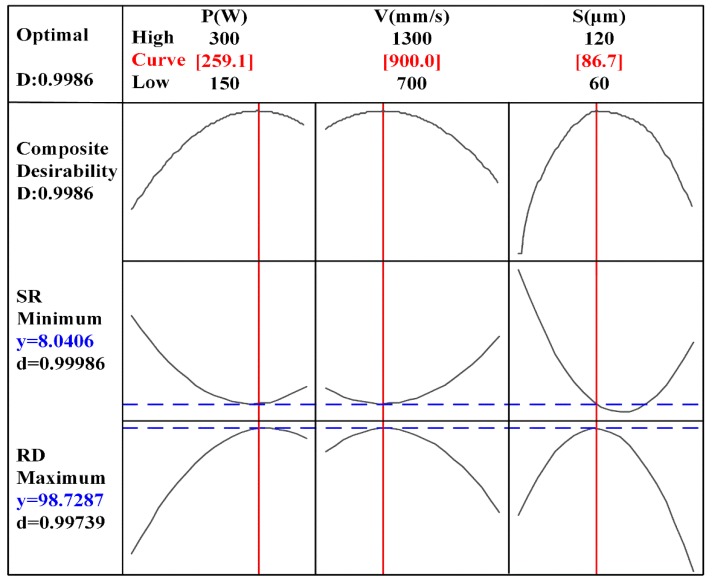
Multi-objective optimization plot for minimizing SR and maximizing RD.

**Figure 6:**
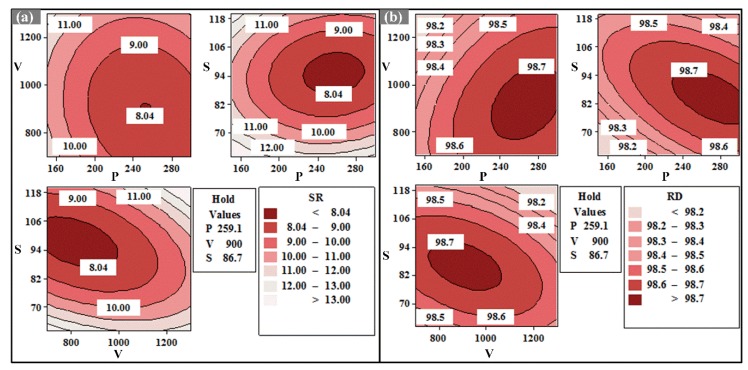
Contour plots of (**a**) SR and (**b**) RD at hold values of P (259.1 W), V (900 mm/s), and S (86.7 μm), respectively.

With the aim of obtaining the maximum RD and minimum SR simultaneously, multi-objective optimization with an equal weight considering both RD and SR were carried out according to the measured results. The optimization result is shown in Figure 5. Apparently, the composite optimal value can be obtained when the processing parameter set (P, V, S) is (259.1 W, 900 mm/s, 86.7 μm), meaning that the RD of 98.72% and the SR of 8.04 μm can reach the relative optimal values simultaneously. Here, it is interesting that the S of 86.7 μm is not the optimal value in the curve for the SR. Based on the composite optimal parameters, the contour plots of SR and RD can be obtained respectively, as shown in Figure 6. The crimson zones represent optimal processing window where optimal response (SR and RD) can be achieved simultaneously. Comparing Figure 6a with Figure 6b, it is obvious that the optimal processing window of RD is larger than that of SR, meaning that the SR is more sensitive to variation of processing parameters and should be given priority in the optimization process. At the same time, it is worth noting that the positions of optimal processing windows for SR and RD are basically the same, suggesting that collaborative optimization of the two responses is effective.

## 4. Discussion

The effects of processing parameters on RD are revealed. For main effects, both P and V have significant effects on the RD of parts, but the effect of S is weaker. This phenomenon can be attributed to the selection of the processing window where the P and the V have formed comparatively optimal processing sets, resulting in the S with a small range (60–120 μm) only having a relatively weak effect on the RD. Below 260 W, P has a positive correlation with the RD, which can be explained by the energy used to melt the powder being directly dependent on the P. Therefore, the higher P is, the higher RD will be. When P is too low, the metal powder cannot obtain enough energy to melt completely, easily causing the balling phenomenon and thus reducing the RD of the parts. Furthermore, too much laser power will induce instability of the molten pool and easily form keyhole defects, causing a drop in RD. In the defined range of V, a lower V is more conducive to obtaining a good RD for parts. It is well-known that the solidification rate of the molten pool is mainly determined by the V. With the increase of V, it is possible that the molten pool has solidified before the powder is completely melted, leading to the decrease of the RD. Additionally, too large or too small S value are both bad for RD. In fact, the S is closely related to the width of the single-track molten pool. If S is too large, the powder between adjacent molten paths is not completely melted, which causes inner porosity and bad RD of parts. If S is too small, there are major overlapping zones between adjacent single-tracks, leading to a large amount of laser energy consumption through thermal conduction of consolidated materials instead of the powder, and causing an uneven temperature field and an unstable molten pool (Figure 4g). For interaction between two factors, it is found that the interaction effects of P*S and V*S are more significant than that of P*V. Therefore, it can be concluded that P and V should be adjusted first in the process of parameter optimization, and then S.

The effects of processing parameters on SR are also illuminated. P and V also have highly significant impacts on the SR of parts, while S has less impact. The results are close to the previous studies about RD, because the surface quality of each layer directly affects the inner porosity (porosity = 1 - RD) of the sample. The interaction effects of SR, P*V and P*S have very weak influences on SR, which can be explained by the decisive effect of P. Changes of V and S do not affect the significance of P on SR. However, the effect of V*S on SR is highly significant, which is due to the S value depending on the influence of V on the single-track width.

When the composite desirability reaches the optimal value, not all parameters are optimal for RD and SR. This phenomenon is attributed to the interaction between processing parameters and compromise between optimization objectives.

## 5. Conclusions

This paper focuses on the multi-objective optimization containing the RD and the SR properties of 316L stainless steel in SLM using RSM. The influences of processing parameters on the RD and the SR were investigated and discussed. The following conclusions can be drawn:

1. For the main effects of single factor, the influences of different processing parameters on the RD and the SR of 316L stainless steel are similar. The effects of P and V on RD and SR of parts are highly significant, but that of S is weak.
2. For interaction effects between two factors, there are some differences between the RD and the SR. All of the interaction influences containing P*V, P*S, V*S on the RD behave significantly, whereas for the SR only the V*S has a significant influence.
3. Based on the RSM and the ANOVA, the mathematical relationship model between the RD/SR and processing parameters have been built, and can be used to effectively predict the processing parameters set or the target response.
4. According to multi-objective optimization, an optimal processing parameters set with (P, V, S) values of (259.1 W, 900 mm/s, 86.7 μm) has been obtained. A resultant high RD of 98.7% and excellent SR of 8.04 μm can be achieved simultaneously using these values, which can further improve fatigue properties of SLMed 316L stainless steel products.

## Figures and Tables

**Table 1:** Machine specifications and parameters.

Property	Value
Machine	FS271M
Platform Dimension (L × W × H)	275 mm × 275 mm × 320 mm
Laser Type	Fiber laser
Laser Diameter	70~200 μm
Maximum Laser Power	500 W
Maximum Scan Speed	15.2 m/s
Layer Thickness	0.02~0.1 mm
Volume Forming Rate	20 cm^3^/h

**Table 2:** Different levels and coded values of processing parameters in the RSM (response surface method).

Input Factors (Coded Values)	The Levels of Input Factors				
	−1.682	−1	0	1	1.682
Laser Power (W)	150	180.4	225	269.6	300
Scanning Speed (mm/s)	700	821.6	1000	1178.4	1300
Hatch Spacing (μm)	60	72.2	80	107.8	120

**Table 3:** Experimental design matrix and measured results of selective laser melted (SLMed) 316L stainless steel parts.

Standard Sequence	The Processing Parameters			Measured Value	Calculated Value	Measured Value
	P (w)	V (mm/s)	S (μm)	Density (g/cm^3^)	RD (%)	SR (μm)
1	180.4	821.6	72.2	7.845	98.31	11.57
2	269.6	821.6	72.2	7.870	98.62	10.38
3	180.4	1178.4	72.2	7.840	98.25	10.90
4	269.6	1178.4	72.2	7.871	98.63	10.35
5	180.4	821.6	107.8	7.869	98.61	10.25
6	269.6	821.6	107.8	7.863	98.53	8.47
7	180.4	1178.4	107.8	7.840	98.25	12.18
8	269.6	1178.4	107.8	7.851	98.38	10.73
9	150.0	1000.0	90.0	7.850	98.37	10.62
10	300.0	1000.0	90.0	7.877	98.71	8.35
11	225.0	700.0	90.0	7.879	98.73	8.11
12	225.0	1300.0	90.0	7.850	98.37	10.26
13	225.0	1000.0	60.0	7.855	98.43	11.73
14	225.0	1000.0	120.0	7.847	98.33	10.94
15	225.0	1000.0	90.0	7.872	98.65	8.73
16	225.0	1000.0	90.0	7.871	98.63	8.41
17	225.0	1000.0	90.0	7.870	98.62	8.06
18	225.0	1000.0	90.0	7.875	98.68	8.35
19	225.0	1000.0	90.0	7.877	98.71	8.04
20	225.0	1000.0	90.0	7.874	98.67	8.06

**Table 4:** Analysis of variance results for RD and SR.

Source	DOF	Sum of Squares		Mean Square		The *F*-Value		*P*-Values		
		RD	SR	RD	SR	RD	SR	RD		SR
Model	9	0.513907	37.2364	0.057101	4.1374	31.42	31.07	0.000		0.000
Linear	3	0.228636	9.9670	0.076212	3.3223	41.94	24.95	0.000		0.000
P	1	0.126006	5.6545	0.126006	5.6545	69.34	42.47	0.000		0.000
V	1	0.099457	3.6973	0.099457	3.6973	54.73	27.77	0.000		0.000
S	1	0.003173	0.6152	0.003173	0.6152	1.75	4.62	0.216		0.057
Square	3	0.197821	23.8852	0.06594	7.9617	36.28	59.80	0.000		0.000
P^2^	1	0.034911	4.0903	0.034911	4.0903	19.21	30.72	0.001		0.000
V^2^	1	0.030076	2.6237	0.030076	2.6237	16.55	19.71	0.002		0.001
S^2^	1	0.161276	20.2991	0.161276	20.2991	88.74	152.45	0.000		0.000
Two-Factor Interaction	3	0.08745	3.3841	0.02915	1.1280	16.04	8.47	0.000		0.004
P*V	1	0.0098	0.1176	0.0098	0.1176	5.39	0.88	0.043		0.369
P*S	1	0.0512	0.2775	0.0512	0.2775	28.17	2.08	0.000		0.179
V*S	1	0.02645	2.9890	0.02645	2.9890	14.55	22.45	0.003		0.001
Error	10	0.018173	1.3315	0.001817	0.1331					
Lack of Fit	5	0.012573	0.9529	0.002515	0.1906	2.25	2.52	0.198		0.167
Pure Error	5	0.0056	0.3785	0.00112	0.0757					
Total	19	0.53208	38.5679							
**Summary of the Model**										
Standard Deviation		Determination Factor R^2^			R^2^ (Calibration)		R^2^ (Prediction)			
RD	SR	RD		SR	RD	SR	RD		SR	
0.0426299	0.364896	96.58%		96.55%	93.51%	93.44%	79.84%		79.87%	
